# Rheb1 is required for limb growth through regulating chondrogenesis in growth plate

**DOI:** 10.1007/s00441-024-03861-2

**Published:** 2024-01-23

**Authors:** Yuwei Zhang, Jiaxin Wen, Ruijun Lai, Jiahuan Zhang, Kai Li, Yue Zhang, Anling Liu, Xiaochun Bai

**Affiliations:** 1 School of Basic Medical Science, Southern Medical University, Guangzhou, Guangdong 510515 People’s Republic of China; 2 The Third Affiliated Hospital of Southern Medical University, Guangzhou, Guangdong 510515 People’s Republic of China; 3 Laboratory Medicine, Guangdong Provincial People’s Hospital, Guangdong Academy of Medical Sciences, Guangzhou, 510000 People’s Republic of China

**Keywords:** Rheb1, Chondrogenesis, Chondrocyte Proliferation, Chondrocyte maturation, mTORC1

## Abstract

Ras homology enriched in the brain (Rheb) is well established as a critical regulator of cell proliferation and differentiation in response to growth factors and nutrients. However, the role of Rheb1 in limb development remains unknown. Here, we found that Rheb1 was dynamically expressed during the proliferation and differentiation of chondrocytes in the growth plate. Given that Prrx1^+^ limb-bud-like mesenchymal cells are the source of limb chondrocytes and are essential for endochondral ossification, we conditionally deleted *Rheb1* using *Prrx1-Cre* and found a limb dwarfism in *Prrx1-Cre; Rheb1*^*fl/fl*^ mice. Normalized to growth plate height, the conditional knockout (cKO) mice exhibited a significant decrease in column count of proliferative zones which was increased in hypertrophic zones resulting in decreased growth plate size, indicating abnormal endochondral ossification. Interestingly, although Rheb1 deletion profoundly inhibited the transcription factor Sox9 in limb cartilage; levels of runx2 and collagen type 2 were both increased. These novel findings highlight the essential role of Rheb1 in limb growth and indicate a complex regulation of Rheb1 in chondrocyte proliferation and differentiation.

**Supplementary Information:**

The online version contains supplementary material available at 10.1007/s00441-024-03861-2.

## Introduction

In vertebrate skeletal development, condensation of mesenchymal stem cells drives endochondral bone formation during limb bud growth. The cells of most condensations differentiated into chondrocytes, which sequentially undergo resting, proliferative, prehypotrophic, and hypertrophic stages before apoptosis (Chagin and Newton 2020; Kronenberg 2003; Olsen et al. 2000; Shimizu et al. 2007). Disarrangement in any stage would result in a failure of endochondral bone elongation causing skeletal dysplasia. Thus, growth plate development provides an ideal model to investigate local signaling that influences chondrogenic fate.

Somatic mesoderm and lateral plate mesoderm are the two populations of embryonic mesoderm which contribute to the skeletal system development (Shimizu et al. 2007). Paired-related homeobox 1 (PRRX1)^+^ limb-bud-like mesenchymal stem cells originate from the lateral plate mesoderm (LPM) (Durland et al. 2008; Prummel et al. 2020) and are the progenitors of Sox9^+^ cells which can direct differentiation towards the chondrogenic lineage (Akiyama et al. 2005; Logan et al. 2002; Shimizu et al. 2007). Thus, Prrx1-expressing cells have been investigated as a cell source to regenerate injured cartilage or fractured bone; however, the regulatory mechanisms that control cell fate in the Prrx1 lineage are incompletely understood.

Rheb, a GTPase belonging to the Ras superfamily of proteins, plays critical roles in cellular and physiological homeostasis (Heard et al. 2014; Saito et al. 2005; Yamagata et al. 1994). The essential function of Rheb in growth regulation in response to nutrients confirms that it has been conserved during evolution (Saxton and Sabatini 2017). In the absence of growth factors, activated TSC acts as a GTPase-activating protein converting Rheb1 from the GTP-bound form to the GDP-bound form, resulting in signaling inhibition of the mammalian target of rapamycin complex 1 (mTORC1) (Duran and Hall 2012; Garami et al. 2003; Li et al. 2004; Yang et al. 2017). One study indicated that deletion of mTOR or Raptor in Prrx1-positive stem cells, while causing mTORC1 inhibition, did not impact on chondrocyte proliferation or survival, nor did it inhibit chondrocyte growth (Chen and Long 2014). We have demonstrated that mTORC1 is a vital signal in parathyroid hormone-related protein (PTHrP) expression during bone development and have shown that constitutive activation of mTORC1 leads to increased chondrocyte proliferation and suppressed chondrocyte differentiation and maturation (Yan et al. 2016). However, accumulating evidence demonstrates that Rheb may function through an mTORC1-independent pathway and that mTORC1 may be activated independently from Rheb. Two studies have shown that Rheb-modulated chondrocytes or adipose-derived mesenchymal stem cells have a potential in cell therapy for the management of cartilage defects (Ashraf et al. 2017a, b), but the role of Rheb in skeletal morphogenesis during bone growth has not been reported.

Since Rheb1 is ubiquitously expressed whereas Rheb2 is primarily expressed in the brain (Saito et al. 2005), in the current study, in order to obtain an insight into the role of Rheb1 in skeletal growth from the current study, we conditionally deleted Rheb1 in Prrx1 + limb-bud-like mesenchymal cells (Logan et al. 2002). Our in vivo data provides evidence to demonstrate the essential role of Rheb1 during the chondrogenic process in the developing limb. Importantly, we reveal that Rheb1 deficiency significantly suppresses chondrocyte proliferation while promoting terminal differentiation, indicating that Rheb1 has a stage-dependent function in regulating chondrogenic fate.

## Materials and methods

### Animals

Prrx1-Cre mice were purchased from The Jackson Laboratory, and the Rheb1-flox mouse line was a generous gift from Dr. Xiao Bo (West China Hospital, Sichuan University, China). To generate limb mesenchyme-specific Rheb1 deletion mice, Rheb1-flox mice were crossed with Prrx1-Cre mice. The Prrx1-Cre; Rheb1^flox/flox^ mice were designated Rheb1-cKO, and the littermates (Rheb1^flox/flox^) were referred to as controls. All animal experiments were approved by the Animal Care and Use Committee of the Southern Medical University (Guangzhou, China).

### Polymerase chain reaction (PCR)

Genotyping was conducted by PCR with DNA samples from mouse tails. The primers used were as follows: Prrx1-Cre-forward 5′-TCCAATTTACTGACCGTACACCAA-3′, Prrx1-Cre-reverse 5′-CCTGATCCTGGCAATTCGGCTA-3′, Rheb1-flox-forward 5′-GCCCAGAACATCTGTTCCAT-3′, Rheb1-flox-reverse 5′-GGTACCCACAACCTGACACC-3′.

### Western blot

Cells and tissues were lysed in 2 × SDS lysis buffer containing phosphatase and proteinase inhibitors. The lysates were centrifuged, and the supernatants were separated by sodium dodecyl sulfate–polyacrylamide gel electrophoresis (SDS-PAGE) and blotted onto a nitrocellulose membrane (Bio-Rad Laboratories). The membranes were then analyzed by using specific antibodies and visualized using an enhanced chemiluminescence kit (ECL Kit, Amersham Biosciences).

### Immunofluorescence (IF) staining

Tissues of mice harvested at different ages were fixed in 4% paraformaldehyde (PFA) overnight at 4 °C, decalcified in 0.5 M EDTA, pH 7.4, on a shaker for 1–3 weeks, and then embedded in paraffin. Immunofluorescence staining was performed on 3 µm paraffin sections. After deparaffinization and rehydration, sections were incubated in citrate buffer (10 mM citric acid, pH 6.0) for 16 h at 60 °C to unmask antigens. Thereafter that, the sections were permeabilized with 0.2% Triton X-100 in PBS for 5 min at room temperature and then blocked with 1% sheep serum at room temperature for 1 h. Then, sections were immunostained with primary antibodies (in 1% BSA, 0.2% Triton X-100) at 4 °C overnight. For secondary reactions, species-matched Alexa Fluor 488- and Alexa Fluor 594-labelled secondary antibodies were used (1:500 in 1% BSA, 1 h) at room temperature in the dark. The sections were mounted with medium containing DAPI (ThermoFisher), and images were captured using a FluoView FV1000 confocal microscope (Olympus).

### Antibodies

The following primary antibodies were used: rabbit anti-RHEB (abcam, ab25873), rabbit anti-Ki67 (CST, 9129), anti-bromodeoxyuridine (BrdU; Sigma, b8434), rabbit anti-pS6 (S235/S236, CST, 4858), rabbit anti-Collagen II (abcam, ab188570), rabbit anti-SOX9 (ABclonal, A19710), rabbit anti-RUNX2 (ABclonal, A11753), and rabbit anti-PCNA (abcam, ab92552).

### Skeletal staining

Seven-day-old mice were euthanatized for whole-mount skeletal staining. Specimens were prepared by removing the skin, organs, and brown fat. They were then dehydrated and fixed in 95% ethanol. For further removal of fatty tissue and tissue permeabilization, specimens were exposed to acetone and then consecutively transferred to Alcian blue (Sigma) and Alizarin red (Sigma) staining solutions. Concurrent with Alizarin red staining, exposure to potassium hydroxide (KOH) hydrolyzed the soft tissue, resulting in transparency and allowing visualization of stained skeletal elements.

### BrdU chase assay

In the BrdU chase assay, 2-week-old mice received two intraperitoneal injections of BrdU (Invitrogen; 1 ml/100 g body weight) with an intervening interval of 6 h and were euthanatized 48 h after the first injection to ensure that BrdU-labelled chondrocytes had sufficient time to differentiate. Long bones were harvested, fixed in 4% paraformaldehyde, decalcified in EDTA, and embedded in paraffin. Visualization of BrdU was performed via immunohistochemistry or immunofluorescence with the anti-BrdU antibody.

### Cell proliferation assay

Cell proliferation was measured with Cell Counting kit 8 (CCK8; abcam), using a 3-(4,5-dimethyl-2-thiazolyl)-2,5-diphenyl-2H-tetrazolium bromide assay according to the product protocol.

### Statistical analysis

All results are presented as the means ± S.D. Curve analysis was determined using Prism (GraphPad). The data in each group were analyzed using unpaired, two-tailed Student’s *t*-tests. A statistical level of significance was set at *P* < 0.05.

## Results

### Rheb1 deletion in limb-bud-like mesenchymal cells produces limb malformation

**Fig. 1:**
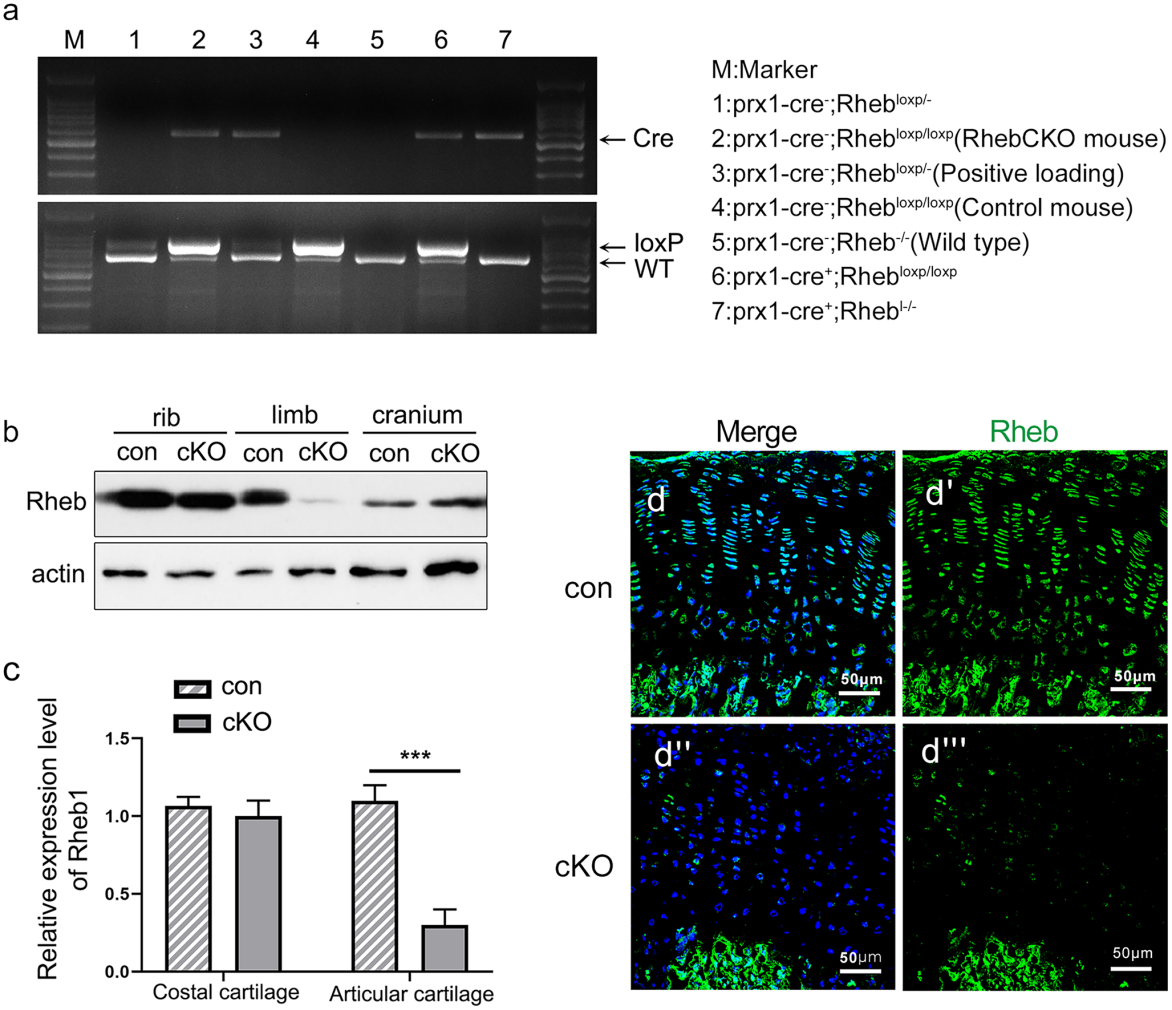
Generation of Rheb1-cKO mice. **a** PCR genotype determination of Rheb1-cKO mice and littermate controls. **b** Western blot analysis of the protein expression level of Rheb1 in ribs, limbs, and craniums from 1-week-old paired mice. *n* = 3. **c** Quantitative PCR analysis of mRNA levels of Rheb1 in costal cartilage or articular cartilage from 2-week-old Rheb1-cKO mice and littermate controls. *n* = 3. **d**–**d’’’** Immunofluorescence staining of Rheb1 expression in growth plates from 1-week-old mice. Scale bar, 200 µm. Student’s *t*-test, *P < 0.05. *n* = 3

In order to explore the in vivo roles of Rheb1 in endochondral ossification, we generated Rheb1 conditional knockout mice in limb-bud-like mesenchymal cells by using the Prrx1-Cre mouse line (Fig. 1a). The genotypes of the offspring indicated that the mice were born at the normal Mendelian ratio, demonstrating that Rheb1 is dispensable for mouse embryogenesis in the limb-bud-like mesenchymal cell. Our data from immunoblotting analysis confirmed that Rheb1 deletion occurs in articular cartilage but not in coastal cartilage or cranial bone in Rheb1-cKO mice (Fig. 1b). Consistent with these findings, the mRNA level (Fig. 1c) and protein level of Rheb1 (Fig. 1d–d’’’**)** was significantly decreased in epiphyseal plate chondrocytes of postnatal (P) day 7 Rheb1-cKO mice compared to controls. Low levels of pS6 (Ser 235/236) (Supplementary Fig. 1a,1b) were detected in the epiphyseal plate of 4-week-old Rheb1 cKO mice compared to controls (Supplementary Fig. 1).

**Fig. 2:**
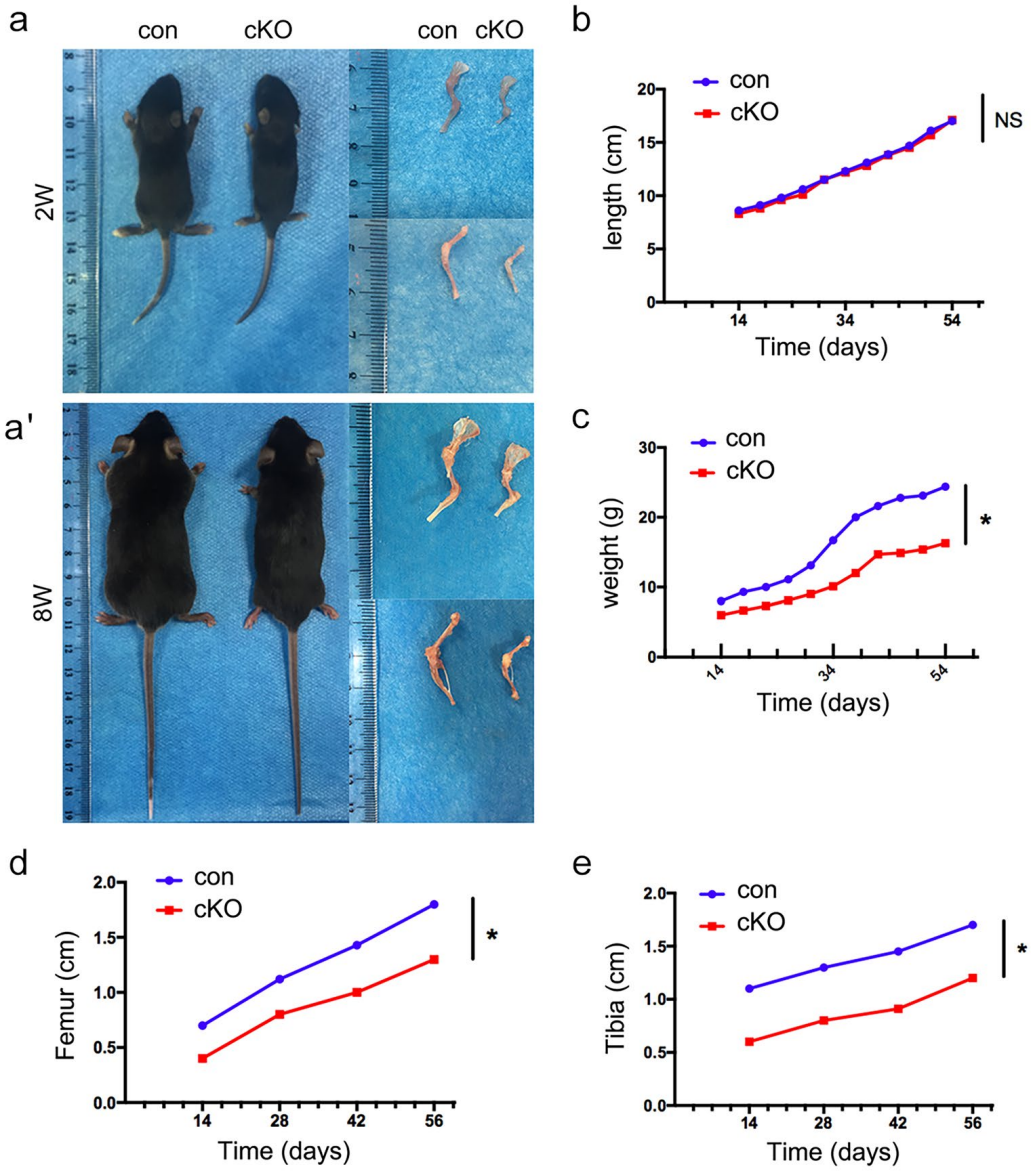
Rheb1 deletion caused limb dwarfism. **a**, **a’** Image of body length and bone length of 2-week- and 8-week-old Rheb1-cKO mice and their littermate controls. Scale bar, 1 cm. Measurement of body length (**b**), body weight (**c**), femur length (**d**), and tibia length (**e**) to reflect the longitudinal growth in Rheb1-cKO mice compared to controls. Student’s *t*-test, *P < 0.05. *n* = 3

The development of long bones involves the formation of a cartilage primordium that is ultimately substituted by a calcified matrix to allow directional growth. In Rheb1-cKO mice, the significant morphological differences in limb growth between the cKO and control mice were observed at P14. Nevertheless, the cKO mice were viable (Fig. 2a, a’). Although the body lengths of the cKO mice were normal (Fig. 2b), the body weights were reduced and the lengths of long bones in the hindlimbs including the femur and tibia were profoundly reduced compared to control littermates (Fig. 2c and d).

### Rheb1 deletion causes increased growth plate height

**Fig. 3:**
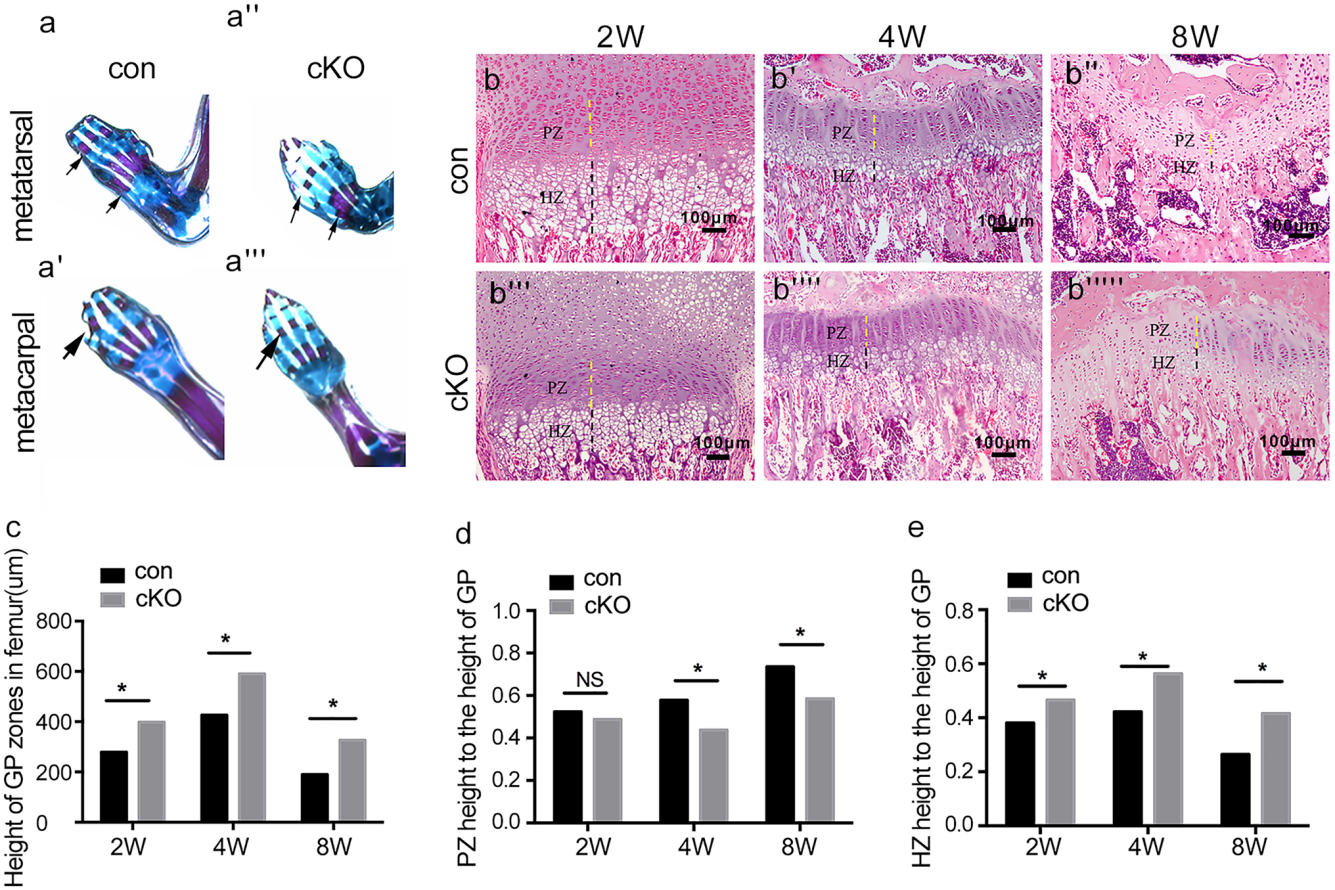
Rheb1 deletion suppressed endochondral ossification. **a** Skeletal preparation by Alizarin red and Alcian blue staining of the 1-week-old Rheb1-cKO mice and littermate controls. Hematoxylin/eosin staining of femur growth plates from 2-week-, 4-week-, and 8-week-old mice (**b**–**b’’’’’**) and quantification of growth (**c**–**e**). Scale bar, 100 µm. Student’s *t*-test, *P < 0.05. *n* = 3

During growth plate development, chondrogenesis occurs as a result of mesenchymal condensation and subsequent processes of differentiation. To characterize the cartilage structure of Rheb1-cKO mice, skeletal preparation by Alizarin red and Alcian blue staining was performed. Strikingly, the appendicular skeleton showed a gross reduction of mineralizing bones (Fig. 3a–a’’’). At the 2-week age, Rheb1-cKO growth plates exhibited an increased proportion height of the hypertrophic zone to the growth plate height but without a detectable difference in the proportion height of the proliferating zone (Fig. 3b–b’’’’’**)**, however, leading to decreased growth plate height (Fig. 3c). At stages of the 4-week and 8-week old, similar defects in the hypertrophic zone were observed and accompanied by decreased proportion height of the proliferating zone relative to the growth plate cartilage (Fig. 3d and e).

### Rheb1 deletion suppresses chondrocyte proliferation while promoting maturation

**Fig. 4:**
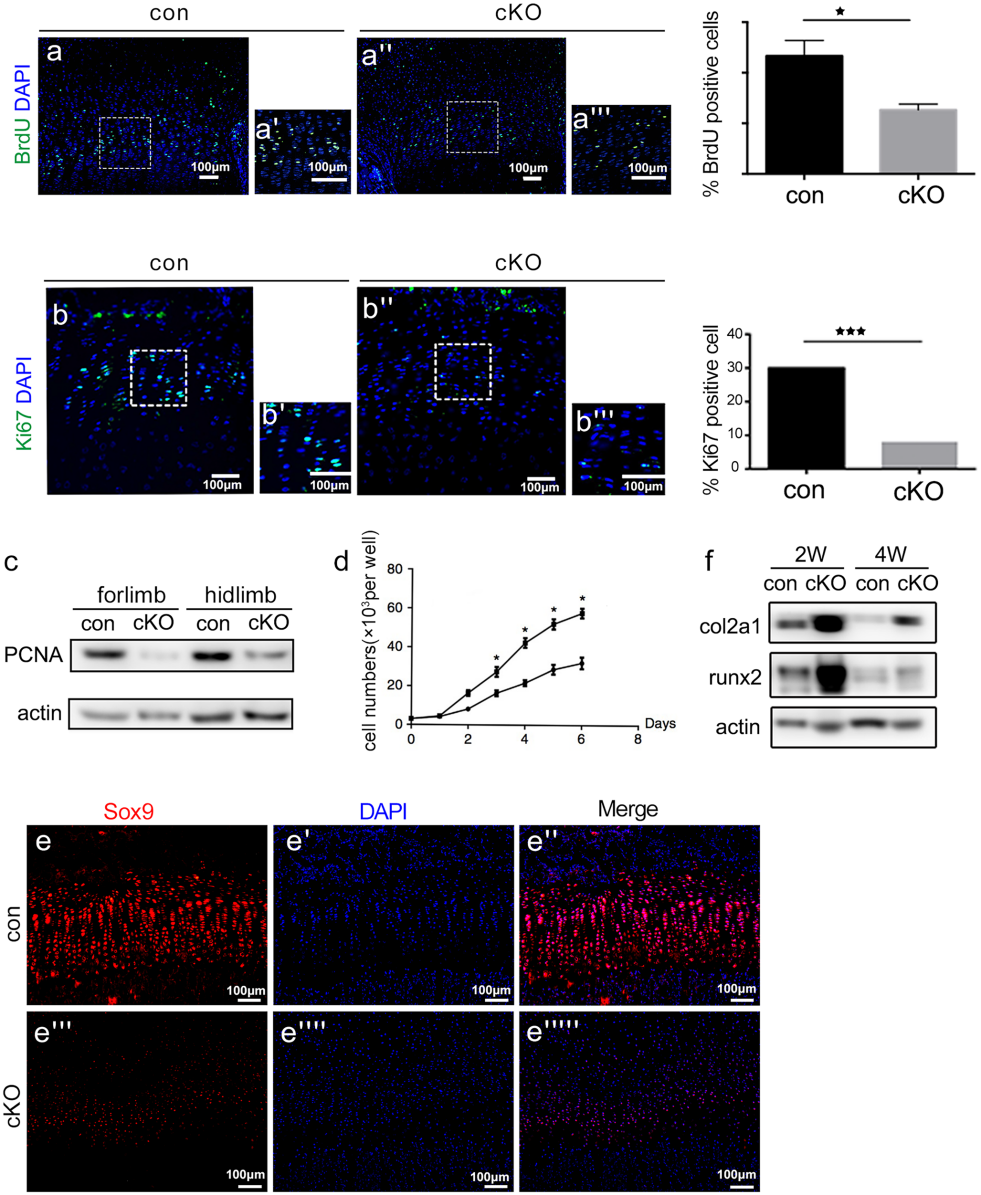
Rheb deletion suppressed chondrocyte proliferation while promoting maturation. **a**–**a’’’** BrdU labelling of chondrocytes in femurs from 7-day-old Rheb1-cKO and control mice. BrdU, green; DNA, blue. Scale bar, 100 µm. BrdU-positive cells were counted. **b**–**b’’’** Immunofluorescence analysis of Ki67-positive cells in femurs from 2-week-old Rheb1-cKO and control mice. Ki67, green; DNA, blue. Scale bar, 100 µm. **c** Immunoblotting analysis showing PCNA levels in limb cartilage from 2-week-old Rheb1-cKO and control mice. **d** CCK-8 proliferation assay of primary chondrocytes isolated from Rheb1-cKO and control mice. OD_450_ values were converted to cell numbers. **e**–**e’’’’’** Immunofluorescence staining of Sox9 in growth plates from 2-week-old mouse femurs. Sox9, red; DNA, blue. Scale bar, 100 µm. **f** Immunoblotting analysis of the protein levels of type 2 collagen and runx2 in limb chondrocytes isolated from Rheb1-cKO and control mice. All data were analyzed by Student’s *t*-tests. Data represent mean values ± SD. *P < 0.05, *n* = 3

Interestingly, we performed a bromodeoxyuridine (BrdU) incorporation assay (Fig. 4a–a’’’) and Ki67 immunofluorescence staining (Fig. 4b–b’’’) to label proliferative cells in Rheb1-cKO mice and found a decrease in chondrocyte proliferation in growth plates of newborn and 2-week-old Rheb1-cKO mice. Consistent with the in vivo data, immunoblotting analysis of limb chondrocytes isolated from newborn Rheb1-cKO mice revealed a significant reduction in proliferating cell nuclear antigen (PCNA) level (Fig. 4c) and proliferative rate (Fig. 4d). On the basis of the increased height proportion of the hypertrophic zone in the cKO growth plate, we hypothesized that except for the proliferative defects, Rheb1-deficient limb-bud-like mesenchymal cells were dysregulated in chondrogenic differentiation or maturation. Therefore, we evaluated the expression levels of chondrocyte differentiation markers and found a significantly decreased expression of Sox9 in Rheb1-cKO cartilage (Fig. 4e–e’’’’’). Immunoblotting analysis revealed pronounced increased levels of type II collagen and runx2 in hindlimb chondrocytes that isolated from Rheb1-cKO mice (Fig. 4f), indicating that chondrocyte differentiation or maturation was promoted.

Taken together, these findings indicate that Rheb1 deleted in limb-bud-like mesenchymal cells was critically involved in limb chondrogenesis during development and played a suppressive role in regulating chondrocyte proliferation while still allowing for chondrocyte maturation and functional protein expression. Thus, using Rheb1-modulated mesenchymal stem cells to repair cartilage damage should be considered a therapeutic approach for functional recovery.

## Discussion

In response to growth factors and nutrients, the mTORC1 signaling is activated by GTP-bound Rheb, subsequently regulating cell growth, proliferation, and differentiation. Our previous studies indicated that activated mTORC1 signaling promotes proliferation but suppresses terminal differentiation of chondrocytes via Gli2-mediated PTHrP expression (Yan et al. 2016). Though Rheb can directly bind to and activate mTORC1 (Li et al. 2010), the specific functions of Rheb in skeletal growth are unclear. Herein, we generated Rheb1 conditional knockout mice using Prrx1-Cre recombinase. Unexpectedly, Rheb1-deficient limb-bud-like mesenchymal cells inhibited proliferation while promoting maturation during chondrogenesis, which resulted in limb shortening. Although the exact molecular mechanisms altering chondrogenic fate were not thoroughly defined in the current study, our investigation has provided to the best of our knowledge the first in vivo evidence for a novel understanding of Rheb1 in regulating limb growth.

Inhibition of mTORC1 in prrx1 + limb-bud-like mesenchymal cells has been shown to suppress chondrocyte differentiation but without affecting proliferation, resulting in reduced cartilage growth. However, in the current study, a significant decrease in chondrocyte proliferation accompanied by an acceleration of chondrocyte maturation occurred in Rheb1-cKO mice. The different cartilage phenotypes observed between Rheb1-deficient mice and mTOR/Raptor-deficient mice were probably caused by (1) Rheb1 regulation of chondrocyte proliferation via both mTORC1-dependent and independent signaling pathways (Karbowniczek et al. 2004; Yang et al. 2021) and (2) a decreased level of PTHrP which is regulated by mTORC1 (Yan et al. 2016) in chondroprogenitors and in turn promotes chondrocyte maturation (Wuelling and Vortkamp 2011). Our findings confirmed that low mTORC1 activity is essential for chondrocyte maturation, but the exacted underlying mechanism requires further investigation.

It has been well established that Sox9 is a pivotal transcription factor in the differentiation of various cell types (Bastide et al. 2007; Mori-Akiyama et al. 2007; Spokony et al. 2002), and accumulating studies have demonstrated that Sox9 is essential for chondrocyte lineage development (Akiyama et al. 2002, 2004; Bi et al. 1999). That chondrocyte proliferation decreased while Sox9 was weakly expressed in Rheb1-cKO mice is consistent with previous ex vivo studies (Ashraf et al. 2017a, b). Remarkably, increased levels of type II collagen and runx2 were observed in Rheb1-deficient limb cartilage. It has been shown that chondrocyte hypertrophy is promoted by enhanced expression of type II collagen (Barry et al. 2001; Lefebvre et al. 1998) and runx2 (Fujita et al. 2004; Yoshida et al. 2004; Zheng et al. 2003), but the opposite effects of Rheb on chondrocyte maturation have been observed between ex vivo (Ashraf et al. 2017a, b) and in vivo experiments, which may be due to a discrepant niche microenvironment in the case of the growth plate (Matsushita et al. 2020).

It is worth noting the expression of prrx1 throughout the early limb bud mesenchyme and in a subset of craniofacial mesenchyme. As can be seen in the mouse images, craniofacial bone development was most likely affected by Rheb1 deletion as reflected by smaller head size, suggesting a possible contribution of Rheb1 in osteogenesis during intramembranous ossification. Thus, further investigations are warranted to clarify whether Rheb is involved in the regulation of chondrocyte-to-osteocyte transformation during endochondral bone formation, physiologically or pathologically.

### Supplementary Information

Below is the link to the electronic supplementary material.

Supplementary file1 (DOCX 6523 KB)

## Data Availability

All data included in this study are available upon request by contact with the corresponding author.
