# Supplementary material for: Rheb1 is required for limb growth through regulating chondrogenesis in growth plate
Source: Cell Tissue Res. 2024 Jan 23;395(3):261–9. doi: 10.1007/s00441-024-03861-2 (PMC10904423; doi:10.1007/s00441-024-03861-2)
Supplement: Supplementary file 1 — Supplementary file1 (DOCX 6523 KB) [file 441_2024_3861_MOESM1_ESM.docx]

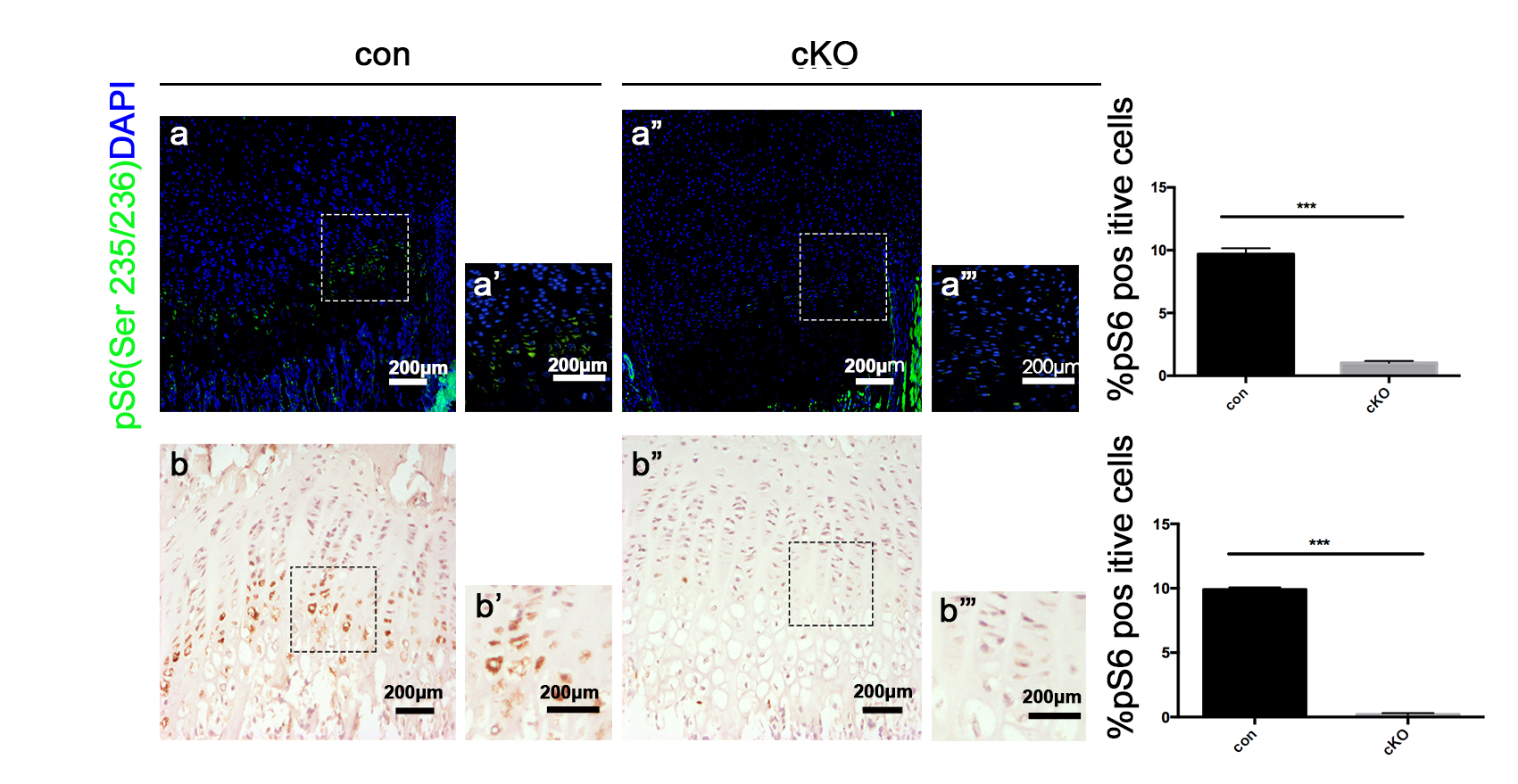


Supplementary Figure1 (a – a’’’) Immunofluorescence staining of pS6 (Ser 235/236) in growth plates from 4-week-old mouse femurs. pS6 (Ser 235/236), green; DNA, blue. Scale bar, 200 μm.(b – b’’’) Immunohistochemistry analysis pS6 (Ser 235/236) in growth plates from 4-week-old mouse femurs. All data were analyzed by Student’s *t*-tests. Data represent mean values ± SD. ***P<0.001.
